# Iliac occlusion due to covered stent deformation following abdominal massages

**DOI:** 10.1016/j.jvscit.2022.02.011

**Published:** 2022-03-09

**Authors:** Gaia Pollorsi, Daniel Danzer, Nicolas Murith, Christoph Huber, Damiano Mugnai

**Affiliations:** a Department of Cardiovascular Surgery, Geneva University Hospital, Geneva, Switzerland; b Department of Vascular Surgery, Wallis Hospital, Sion, Switzerland

**Keywords:** Iliac arteries, Neurogenic bowel, Stents

## Abstract

Extrinsic compression is a potential cause of stent failure. We have described the case of a 65-year-old paraplegic patient with acute right leg ischemia. His medical history was relevant for aortobifemoral bypass, followed by kissing covered stent reconstruction of a proximal anastomotic false aneurysm. The computed tomography scan showed collapse of the right iliac covered stent with ipsilateral iliofemoral graft thrombosis and partial collapse of the left iliac covered stent. He underwent emergent right iliac limb open thrombectomy and redo covered stent relining. The cause of compression was found to be daily deep abdominal massages for intestinal evacuation. The endovascular device should be tailored to the patient's particularities.

Neointimal hyperplasia remains the first cause of long-term stent patency failure.^1^ Stent injury due to excessive lower limb movement, extrinsic compression, or trauma are less reported potential causes of stent occlusion.^2^ To the best of our knowledge, the present report is the first case of stent trauma due to patient self-care. We have described the case of a paraplegic patient with acute limb ischemia caused by iliac balloon expandable covered stent collapse after abdominal massage. Our patient provided written informed consent for the report of his case details and imaging studies.

## Case report

The present patient was a 65-year-old paraplegic man who had been admitted to our emergency department with acute right leg ischemia. His medical history was remarkable for polyaneurysmal disease treated with an aortobifemoral Dacron bypass graft with an end-to-end proximal anastomosis and an end-to-side distal anastomosis in the late 1990s.

To treat an infrarenal aortic anastomotic false aneurysm in our patient, who was considered unfit for surgery, kissing balloon expandable covered stent (10 mm diameter and 57 mm length) reinforcement with proximal flaring to obtain a good seal had been performed a few months earlier. He had also undergone multiple abdominal surgeries, including cystectomy with construction of a Bricker bladder, intestinal resection, and colostomy.

**Fig 1:**
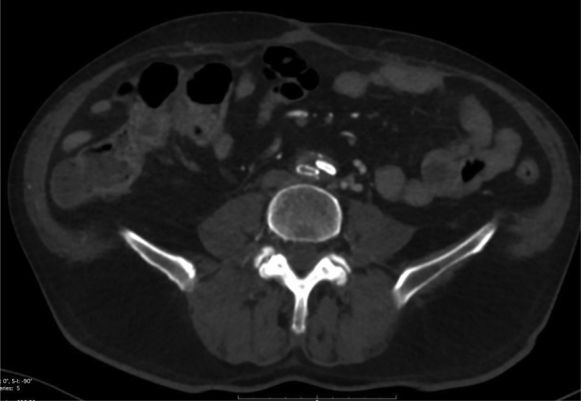
Computed tomography scan showing the right stent crushed and occluded and the left stent partially collapsed but patent.

**Fig 2:**
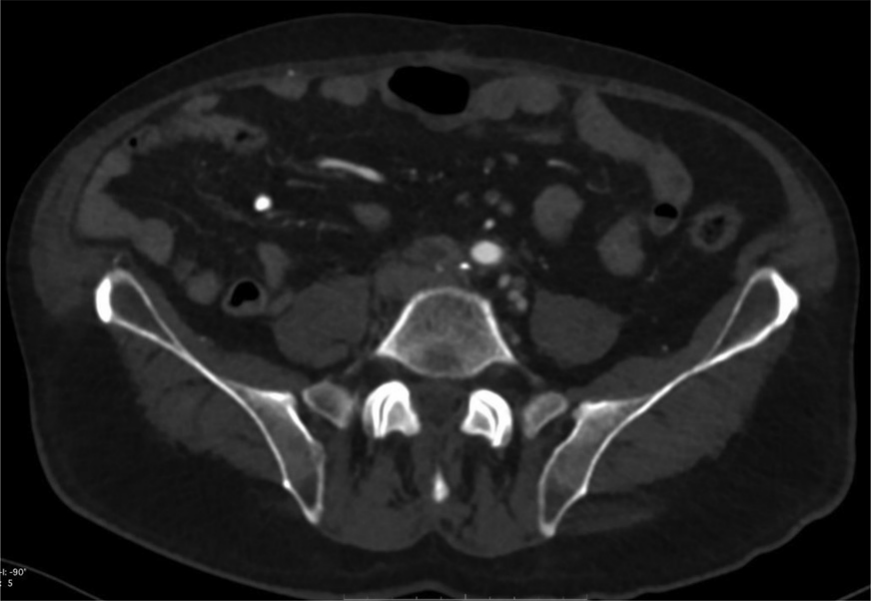
Computed tomography scan showing occlusion of the right stent.

**Fig 3:**
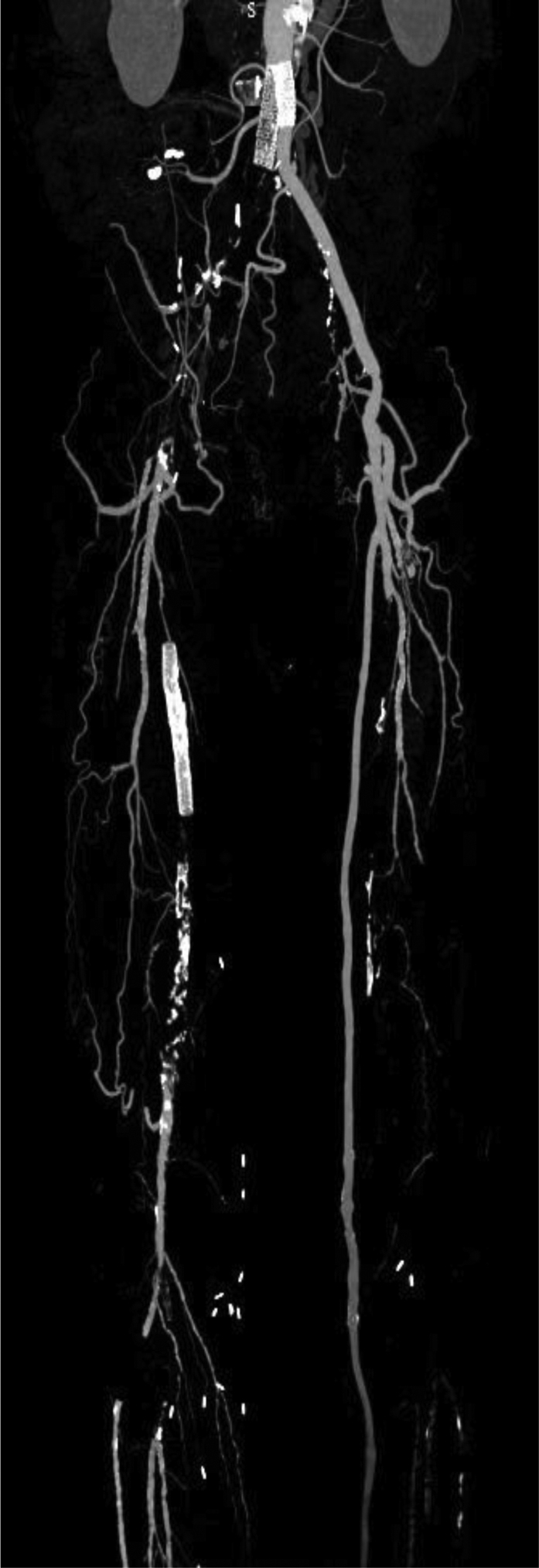
Scan showing reconstruction.

The emergency contrast-enhanced computed tomography scan showed complete exclusion of the false aneurysm, resulting in a collapse of the iliac covered stent and iliofemoral graft thrombosis on the right side and collapse of the iliac covered stent on the left side (Fig 1, Fig 2, Fig 3). The patient underwent emergent right iliac limb thrombectomy through an open femoral approach and percutaneous access on the left side. Intraoperative angiography demonstrated that both iliac stents had been crushed and required bilateral new balloon expandable covered stent implantation. No significant inflation resistance recoil was detected during deployment that was suggestive of extrinsic compression. The postoperative course was uneventful, and the patient was discharged 1 week after surgery with dual antiplatelet therapy.

The crushed stent could not be explained by any anatomic or neoplastic compression. The patient had no history of abdominal trauma. However, both stents had collapsed. After an extensive discussion with the patient regarding his specific conditions, an unexpected etiology was found. Our paraplegic patient had been performing strong abdominal massages starting from the epigastric region, circularly, and descending to the lower abdomen.

The massages had been performed daily for intestinal and bladder evacuation, compressing the periaortic area. Postoperatively, we understood how the stent collapse had occurred, because the patient showed us exactly what he had done and the strong force he had applied. We spoke with him and the nurses and colleagues who specialize in paraplegic patient management, and they confirmed that the abdominal massage had been too intensive. This force was probably sufficient to compress the aortoiliac stents inside because of the patient's thinness.

## Discussion

Because patients with neurologic paraplegia are prone to impaired bowel and bladder function, they must often support visceral mobility with abdominal massage, usually with associated laxative drug use to develop an effective bowel routine.^3^ In our normal-weight patient with compromised sensitivity, the repetitive abdominal massage had progressively crushed both stents against the underlying spine until stent occlusion had occurred. Moreover, the progressive flow impairment had remained asymptomatic until complete occlusion had developed owing to his neurologic impairment.

Because of the short distance between the renal arteries and the prosthetic bifurcation, no conventional aortic stent graft could fit; thus, the use of a double barrel autoexpandable covered stent implantation would have had a high risk of a type I endoleak owing to the magnitude of the discrepancy between the native aortic diameter and the prosthetic iliac limbs. The use of a balloon expandable stent in the present case was intended to create sealed coverage of the pseudoaneurysm by flaring the stent in a covered endovascular reconstruction of aortic bifurcation-like fashion, which would not have been possible using a self-expandable covered stent. Balloon expandable stents are commonly used in the iliac axis because of their relatively high radial resistance, precision, and flaring proprieties, with few mechanical complications.^4^, ^5^, ^6^, ^7^

Ideally, for the present patient, we would have used a covered stent with the characteristics of both a balloon stent and a self-expendable covered stent. However, such a stent does not exist. The patient was well known for having an abdomen with a hostile anatomy owing to the aortobifemoral bypass, Bricker bladder reconstruction, and colostomy. Open revascularization could have resulted in potentially lethal complications, including intestinal, urinary, and infectious complications. The present patient should have received extensive education regarding bowel evacuation management, especially regarding the risks involved with external manual compression. This stent failure etiology is uncommon and very interesting, because we could not find any similar description in reported studies.

## Conclusions

In clinical practice, the use of a covered balloon expandable stent has shown favorable long-term results.^8^^,^^9^ Because the proximal aortoiliac segment is usually not submitted to significant flexion or external intermittent compression, the current practice has been to use a balloon expandable stent in this location. Nevertheless, the daily abdominal massage had crushed both stents in our patient. Therefore, the specific characteristics of each patient and the specific care required should be considered before stent implantation.^10^ Abdominal massage for spinal paraplegic patients should be recognized as a potential cause of stent failure and should be considered during optimal stent selection, with modification of the abdominal massage routine to avoid this complication in this population. Therefore, if balloon expandable stents are required, such as in our patient, we would suggest ensuring that the patient performs the intestinal evacuation correctly.

Stent selection is one of the keys for good long-term endovascular results. Not only the anatomic characteristics, but also the patient's specific individual condition, should be considered when choosing the correct device.
